# Release and degradation of dissolved environmental RNAs from zebrafish cells

**DOI:** 10.1080/15476286.2025.2486281

**Published:** 2025-04-01

**Authors:** Zhongneng Xu, Shuichi Asakawa

**Affiliations:** a Department of Ecology, Jinan University, Guangzhou, China; b Department of Aquatic Bioscience, Graduate School of Agricultural and Life Science, The University of Tokyo, Tokyo, Japan

**Keywords:** Dissolved environmental RNAs, RNA release, RNA degradation, zebrafish cells, fish RNA

## Abstract

The sources and degradation profiles of dissolved environmental RNAs from fish in water remain unknown. In this study, laboratory experiments and mathematical modelling were conducted to investigate the permeability of RNA extracted from zebrafish cells through filters, the release of dissolved environmental RNAs from live and dying zebrafish cells, and the degradation of RNA extracted from zebrafish cells in a non-sterile aqueous environment. This research aimed to provide biological and ecological insights into fish RNAs dissolved in water. The results showed that most of the RNA extracted from zebrafish cells was detected in the filtrates after passage through 0.45 µm filters. Over the course of the 6-day experiment, dynamic levels of the RNAs in the liquid environment containing live or dying zebrafish cells were determined. The release and degradation rates of dissolved environmental RNA from zebrafish cells were calculated using mathematical modelling. RNA extracted from zebrafish cells degraded in non-sterile water in the tubes, and after 2 months, more than 15% of the RNAs in the water remained detectable. The half-life of the RNA in the tubes was approximately 20 ~ 43 days. The modelling results suggest that the levels of the dissolved environmental fish RNAs in natural waters or aquariums could be so low that it would be difficult to detect them using current techniques. The results obtained in this study will help develop new methods for measuring the dynamics of dissolved environmental fish RNAs in water and determining their significance.

## 1. Introduction

There are fish RNAs in water environments, and the detection of environmental RNAs in water has been carried out in studies of fish biology and fish ecology, such as fishery resource surveys, fish biodiversity surveys, water pollution monitoring, and analyses of the living state of fish [1–6]. Although aquatic environment RNA has great potential for application, there are still some technical limitations. By filtering water samples, environmental RNAs in water can be divided into particulate environmental RNAs and dissolved environmental RNAs. Particulate environmental RNAs in water are those that remain on the filters, probably comprising RNA transcripts in cells and released tissues. Dissolved environmental RNAs in water are those found in the filtrates, primarily released by cell rupture or secretion from cells. Current filtration methods for detecting environmental RNAs in water involve separating the RNA in a water sample into particulate environmental RNA, which is attached to the filter membrane, and dissolved environmental RNA, which is found in the filtrate. These methods primarily measure particulate RNAs that remain on the filters with different pore sizes, such as 0.2–0.22 µm [7–9], 0.45 µm [2–4,10–12], 0.7 µm [5,13–15], 1.2 µm [11,16], 1.6 µm [17], and 5 µm [11]. However, these methods do not measure the amount of dissolved RNA in the filtrates, making it unclear how much environmental fish RNA passes through the filters.

Previous studies have shown that the content of total dissolved RNA in natural water bodies is extremely low and cannot even be detected in some water samples [18–24]. As a result, it may be challenging to measure fish dissolved environmental RNA in water bodies. Currently, basic information about fish dissolved environmental RNA – such as the production and degradation processes – has not been reported. Studies have indicated that dead cells can leak RNAs into the external environment and live mouse embryonic fibroblast 3T3 cells can also release RNA [25,26]. However, the origin and composition of the fish RNAs detected in aquatic environment – whether it originates from live, dead or both types of fish cells – remains unclear. Moreover, the half-life of the environmental fish RNAs dissolved in water is still unknown.

While it is difficult to measure fish dissolved environmental RNA in natural environments and aquariums, we successfully obtained fish dissolved RNA in laboratory-controlled conditions. By studying these fish-derived dissolved RNAs, we can infer and estimate certain aspects of fish dissolved environmental RNA in water, providing a foundation for further research on fish dissolved environmental RNA in both wild environments and aquariums. In this study, three laboratory experiments, coupled with mathematical modelling, were conducted to explore the dynamics and sources of fish dissolved environmental RNAs in water: (1) characterization of RNA extracted from zebrafish cells through filters, (2) release of dissolved environmental RNAs from live and dying zebrafish cells, and (3) degradation of RNA extracted from zebrafish cells.

## 2. Results

### 2.1. RNA extracted from zebrafish cells after filtration through a 0.45 µm filter

**Figure 1.**
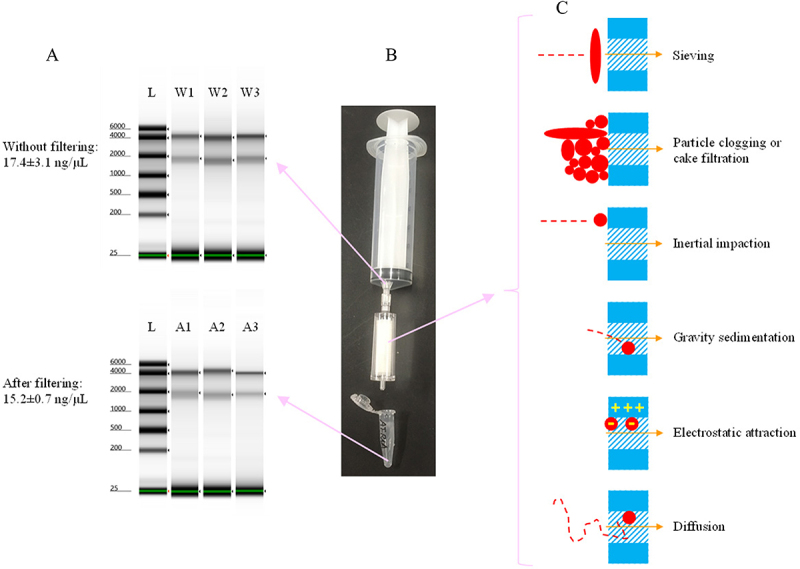
RNA extracted from zebrafish cells through a 0.45 µm filter. a. Gel electrophoresis results of RNA analysed by the RNA ScreenTape. L represents the ladder. W1, W2, and W3 represent the replicates of the original samples without filtration. A1, A2, and A3 represent the samples after filtering with 0.45 µm filter units. b. Apparatus used for filtering liquid samples and collecting the filtrates. A needleless syringe was used to force the liquid sample into the filter unit, and the filtrate is collected in a centrifuge tube after filtration. The pore size of the filter unit is 0.45 µm. c. Schematic illustrating potential mechanisms by which RNAs remain on the filters. The red solid circles and the red solid ellipses represent RNAs. The red dashed path represents the movement trajectory of the RNA. The blue shaded area represents the filter pore, and the unshaded blue area represents the filter wall. The orange arrow indicates the direction of liquid flow.

Following filtration with 0.45 µm filters (Figure 1), only 13% of the total RNA was retained on the filter, while the remaining 87% of the total RNA passed through. Approximately 70% of 18S RNA and about 84% of 28S RNA passed through the filter. Various factors, such as sieving, particle clogging, and electrostatic attraction [27,28], could affect the RNA retention on the filter (Figure 1(C)). These results suggest that the filter method is insufficient for collecting environmental RNAs, including both particulate environmental RNAs and dissolved environmental RNAs.

### 2.2. Dissolved environmental RNAs released by live and dying zebrafish cells

**Figure 2.**
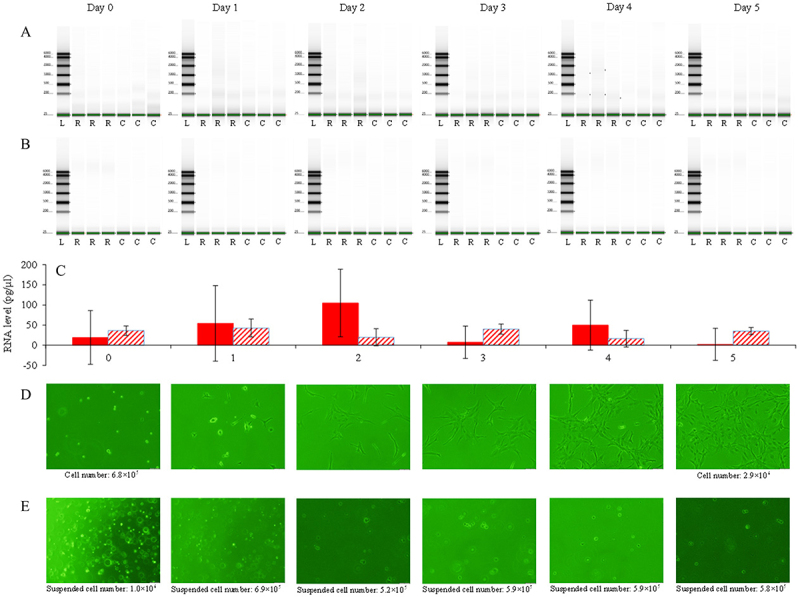
Dissolved RNAs in the liquid environment released by live and dying zebrafish cells during the 6-day experimental period. a. Level of the dissolved RNA in the medium of cultured zebrafish BRF41 cells: results from TapeStation detection. L represents the ladder. R represents the dissolved RNA in the L-15 medium in which the zebrafish BRF41 cells are cultured. C represents the L-15 medium control. b. Level of the dissolved RNA in the nuclease-free water containing the zebrafish BRF41 cells: results from TapeStation detection. L represents the ladder. R represents the dissolved RNA in the nuclease-free water in which the zebrafish BRF41 cells are cultured. C represents the nuclease-free water control. c. Average level of dissolved RNA in the liquid environment released by live and dying zebrafish cells. The red solid column represents the average level of dissolved RNA in the L-15 medium released by the live zebrafish BRF41 cells, and the black bar indicates the standard deviation. The red shaded column represents the average level of the dissolved RNA in the nuclease-free water released by the dying zebrafish BRF41 cells, with the black bar indicating the standard deviation. d. The physiological state of the zebrafish BRF41 cells cultured in L-15 medium. On day 0, the cells are suspended in the medium and counted by sampling. On days 1, 2, 3, and 4, the cells are attached to the bottom of the flasks and could not be counted. On day 5, the cells are dislodged by using the trypsin and then counted. e. The physiological state of the zebrafish BRF41 cells in nuclease-free water. No cells are attached to the bottom of the flasks within the 6-day period.

**Figure 3.**
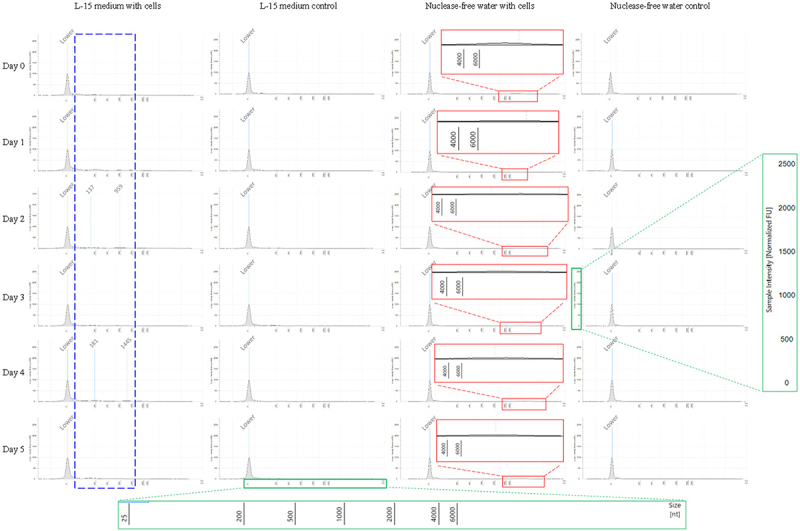
The sizes of the dissolved RNAs released by live and dying zebrafish cells during the 6-day experimental period. The results are measured using the TapeStation (high sensitivity RNA ScreenTape). The blue dashed squares represent the primary sizes of the dissolved RNAs in the L-15 medium used to culture the zebrafish cells. The red solid squares represent the primary sizes of dissolved RNAs in nuclease-free water containing zebrafish cells. The green solid squares represent the large font sizes of the coordinate axes in the original images.

Dissolved RNAs released from live zebrafish cells in L-15 medium and from dying cells in nuclease-free water were detected (Figures 2(A,B,C)). During the 6 days of the experiment, fluctuations were observed in the levels of dissolved environmental RNAs released by both live and dying zebrafish cells (Figure 2(C)). The dissolved RNAs released by the live zebrafish cells were primarily less than 2000 bases in length, whereas those from the dying zebrafish cells were primarily longer than 4000 bases (Figure 3).

The cells grew well in the L-15 medium, with most of the cells attached to the bottom of the flasks. On day 1, the cells stretched, and their number increased by more than three times during the experiment (Figure 2(D)). However, the cells were considered to be dying in nuclease-free water, with almost no cells attached to the bottom of the flasks, no cell growth was observed during the experiment, and abnormalities in cell morphology were noted starting on day 3 (Figure 2(E)).

### 2.3. Degradation process of RNA extracted from zebrafish cells in a non-sterile aqueous environment

**Figure 4.**
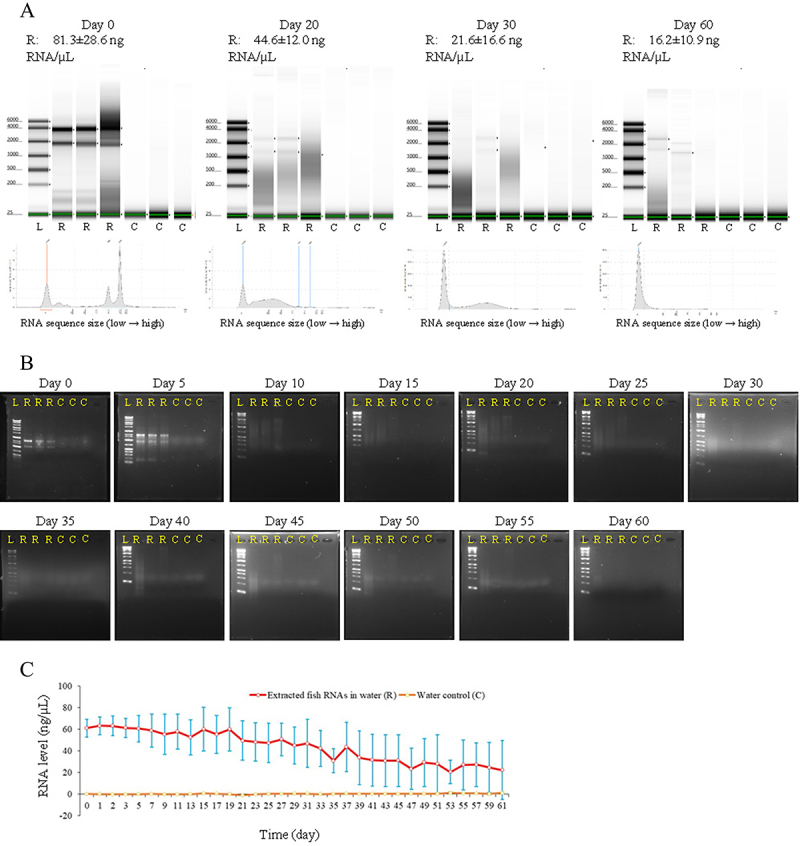
Degradation process of RNA extracted from zebrafish cells in a non-sterile aqueous environment. a. Results from the TapeStation system analysis. L represents the ladder, R represents the extracted zebrafish RNAs in water, and C represents the water control. Samples of Day 0, stored at − 80°C, are analysed on Day 20. b. Results from normal gel electrophoresis analysis. L represents the ladder, R represents the extracted zebrafish RNAs in water, and C represents the water control. c. Results from NanoDrop spectrophotometry. The solid red curve represents extracted zebrafish RNAs in water, the dashed orange curve represents the water control, and the blue bar represents the standard deviation.

The degradation of RNA extracted from zebrafish cells in a non-sterile aqueous environment in tubes was measured using the TapeStation system, gel electrophoresis, and NanoDrop (Figure 4). After two months, more than 15% of the RNAs in the water remained detectable. Over time, RNAs with longer sequences were broken down into short- and intermediate-sized RNA fragments, and by the end of the experiment, the detectable RNAs in the samples were almost all short fragments (Figures 4(A,B)). The half-life of RNAs longer than 25 bases in the tubes was approximately 20 ~ 30 days (Figure 4(A)), and the half-lives of all the RNAs in the tubes were approximately 35 ~ 43 days (Figure 4(C)).

### 2.4. Estimation of the release and degradation of dissolved environmental RNA from zebrafish cells using mathematical modelling

**Figure 5.**
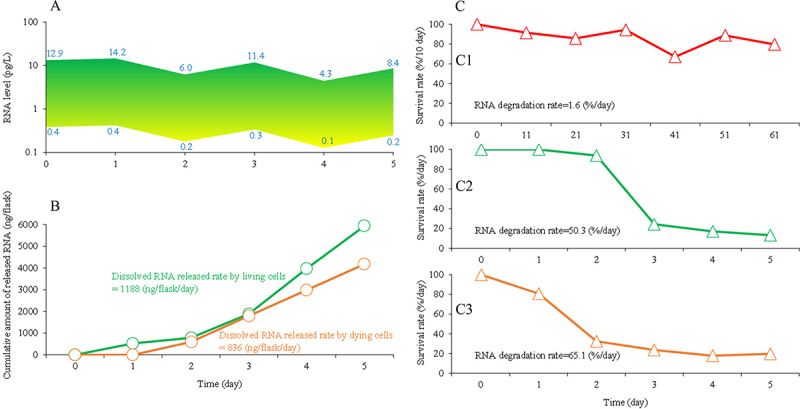
Estimation of the release and degradation of dissolved environmental RNA from zebrafish cells by modelling. a. Estimation of dissolved environmental RNA levels in a fish tank after zebrafish removal. Green represents higher RNA levels, yellow represents lower RNA levels. The number at the top indicates the highest RNA level at the corresponding time point, while the number at the bottom represents the lowest RNA level at the same time point. b. Estimation of the cumulative amount of dissolved RNA released by zebrafish cells. The green curve represents the cumulative amount of dissolved RNA released by live zebrafish cells in L-15 medium. The brown curve represents the cumulative amount of dissolved RNA released by zebrafish cells in nuclease-free water. c. Comparison of estimated degradation rates of dissolved environmental RNAs under different conditions. (c1) No cells: RNA in water in tubes. The red hollow triangle represents the survival rate per 10 days. Data from the experiment on RNA extracted from zebrafish cells in a non-sterile environment (see section 2.3 and section 5.5). (c2) live cells: RNAs in flasks with zebrafish cells in normal medium. The green hollow triangle represents the survival rate per day. Data from the experiment on dissolved environmental RNAs released from the live zebrafish cells (see section 2.2 and section 5.3), and the study of estimation of the release and degradation of dissolved environmental RNA from zebrafish cells by mathematical models (see section 2.4 and section 5.6.2). (c3) dying cells: RNAs in flasks with zebrafish cells in nuclease-free water. The brown hollow triangle represents the survival rate per day. Data from the experiment on dissolved environmental RNAs released by zebrafish cells in nuclease-free water (see section 2.2 and section 5.4), and the study of estimation of the release and degradation of dissolved environmental RNA from zebrafish cells by mathematical models (see section 2.4 and section 5.6.2).

The concentrations of dissolved environmental zebrafish RNA in the water of the zebrafish tank were estimated based on the results from the experiment on dissolved environmental RNAs released by the dying zebrafish cells in nuclease-free water. The results showed that, at a density of three zebrafish per litre of water, the estimated lowest and highest levels of dissolved environmental RNAs in the water were 0.1 pg/L and 14.2 pg/L, respectively (Figure 5(A)).

The estimated amounts of dissolved environmental RNAs released by zebrafish cells are shown in Figure 5(B). The release rate of dissolved RNA by live cells (the cells attached to the bottom of the flasks, with the number of cells increasing by more than three times during the experiment) was greater than that by dying cells (where the cells did not attach to the bottom of the flasks, and the ruptured cells were observed). Environmental RNAs from zebrafish cells dissolved in water were degraded faster in the presence of zebrafish cells (Figure 5(C)). In the absence of zebrafish cells, the survival rates over 10 days were relatively stable at 67–92% over 10 days (Figure 5(C1)). In conditions where live zebrafish cells were present, the degradation of environmental zebrafish RNAs was low during the first 3 days but increased sharply after day 3 (Figure 5(C2)). In the presence of dying zebrafish cells, the environmental zebrafish RNAs degraded gradually during the first 3 days, after which the survival rates remained stable (Figure 5(S3)).

## 3. Discussion

### 3.1. Size of the environmental fish RNAs dissolved in water

According to the present study, fish RNAs of different sizes existed in the water as dissolved environmental RNAs. Environmental RNAs dissolved in water may include functional RNAs, such as mRNAs, short noncoding RNAs, and long noncoding RNAs [29,30]. Therefore, it is theoretically possible that the full RNA complement may be secreted into the water samples, albeit at very low levels. In this study, differences in RNA sizes measured by the TapeStation system from those secreted by live monolayer cells, compared to those secreted from cells transferred to water, suggested differences in the mechanisms of release. However, because whole-transcriptome characterization was not performed in this study, further research is necessary. If the fish RNA remained in the water for a longer time, the environmental fish RNAs that were detectable in the water were shorter in length and contained small amounts of RNA degradation fragments (Figure 4). For dissolved environmental RNAs that are widely present in natural water bodies, data on their RNA degradation fragments may provide important biological and ecological information. Sequencing of the environmental fish RNAs dissolved in water, which may be present in extremely trace amounts, should be developed, such as attempting to reverse transcribe and amplify these dissolved environmental RNAs and then performing whole transcriptome sequencing. However, even in current single-cell transcriptome sequencing technologies that can perform trace RNA sequencing, a cell contains about 10 pg of RNA, while the dissolved environmental fish RNAs in the water were inferred to be 0.1–14.2 pg/L (Figure 5(A)) in this study. Therefore, it is a great challenge to sequence the environmental fish RNAs dissolved in water.

The persistence length of RNA depends on several factors. For example, there is an increase in persistence per base pair of 0.25–0.28 nm, combined with an effective hydrodynamic diameter of approximately 3 nm of a helical RNA, and the flexibility of transient changes in molecular conformation may make the actual length of the RNA longer or shorter [31–33]. Based on the prior literature, which reported RNA’s remaining on filters in size ranges >0.2 µm [7–9], the current study used pore sizes of 0.45 µm filter units. In theory, RNAs of all lengths can pass through pores larger than 0.2 µm in a certain conformation because they are only 3 nm in hydrodynamic diameter. However, in the present study, approximately 13% of the extracted RNAs remained on the filters after filtration (Figure 1(A)). One study showed that the RNA of *Alexandrium pacificum* could be partially collected in filters with pore sizes of 0.45–5 µm [11]. The persistence length of RNAs larger than the pore size, such as RNAs with more than 2000 nucleotides (0.50–0.56 µm in the persistence length), was one of the possible reasons why the filters could collect naked RNAs. Moreover, RNAs with persistence lengths shorter than the filter pore size may remain on the filter membrane through particle clogging, inertial impaction, gravity sedimentation, electrostatic attraction, affinity, and diffusion [20,27,28], as shown in Figure 1(C). It is necessary for us to further investigate whether the RNA retained on the filters had different properties distinct the RNA that passed through the filtrates.

### 3.2. The release of dissolved environmental RNAs by live and dying fish cells

The detection of dissolved RNAs in the medium from cultured zebrafish cells indicated that live fish cells can release dissolved environmental RNAs. There have been some studies on the secretion of RNA by cells: RNA signals have been detected in the medium used to culture mouse embryonic fibroblast 3T3 cells [25]. RNAs can be secreted within extracellular vesicles as carriers for the purpose of cell‒cell communication [34–36]. Additionally, RNAs can be secreted by binding to other macromolecules [37]. These findings support the results of the present study. According to the modelling results, we estimated that an average of 0.41–1.7 pg of dissolved RNA was secreted per cell per day into the liquid environment. If a single nucleotide in RNA is 325 g/mole on average, during the experiment, an average of 7.6 × 10^8^ − 3.2 × 10^9^ RNA bases was secreted per cell per day into the liquid environment. We divided the cumulative amount of released RNA (Figure 5(B)) by the total number of live cells (Figure 2(B)) and estimated that an average of 0.41–1.7 pg of dissolved RNA (approximately 1.7–7.2% of the total amount of cellular RNA) was secreted per cell per day into the liquid environment. Therefore, there may be rich biological information in these bases.

Zebrafish cells in nuclease-free water were regarded as dying cells because, in nuclease-free water, the cells did not attach to the bottom of the flasks, and ruptured cells were observed. The daily amount of environmental RNA released by dying cells increased from the low-release period to the high-release period (Figure 5(B)). In necrosis, a type of cell death, when a cell is subjected to environmental damage, its cell membrane may initially lose permeability, and after certain cell biological processes, the cell becomes leaky, releasing its contents into the surrounding environment [26]. The process of necrosis was consistent with the results of RNA release by zebrafish cells in nuclease-free water. Dying zebrafish cells released lower amounts of dissolved RNA than did the live zebrafish cells. One possible reason for this is that live zebrafish cells absorb nutrients from the medium, convert them into RNAs, and subsequently release more dissolved environmental RNAs than dying cells do.

In the experiments on the dissolved environmental RNAs released by dying zebrafish cells, we did not measure the number of cells that died each day. This made it difficult to further analyse how much of the RNA in nuclease-free water came from dying cells and how much came from surviving cells. The number of cells in the experiment on the dissolved environmental RNAs released by live zebrafish cells increased during the experiment, whereas the number of the dissolved environmental RNAs released by dying zebrafish cells decreased, which may affect the comparison of the results from these two experiments. Consequently, the use of appropriate methods for quantifying live and dead cells, such as flow cytometry, should be considered in future studies.

Using the present experimental results of the dissolved environmental RNAs released from zebrafish cells, fluctuations in the dissolved environmental RNA concentration of 0.1–14.2 pg/L in a fish tank could be calculated (Figure 5(A)). Further work would be necessary to confirm these calculations, including the application or development of techniques that can quantify such low concentration ranges. This calculation of environmental RNAs dissolved in water may differ from that in real bodies of water due to the lack of the specific impacts of the complex physical, chemical, and biological factors in the field.

### 3.3. Degradation of dissolved environmental RNAs in water

The half-life of RNA in cells is typically a few minutes to 2 h in non-mammalian cells and a few minutes to 48 h in mammalian cells [38–43]. In the experiment on the degradation process of RNA extracted from zebrafish cells in water, some RNAs in the water could still be detected after 2 months in the tubes (Figure 2). The half-life of zebrafish RNA in water was 20–30 days, as detected by the TapeStation system, which is much longer than the half-life of RNAs inside bacterial, fungal, and mammalian cells described above. Cellular RNAs that survive for longer periods than normal and accumulate in excess are harmful to cells. Therefore, after RNA is transcribed, its lifespan is controlled by the RNA surveillance system and degradation system [44–46]. The related factors include endoribonucleases, exoribonucleases, the TRAMP complex, Up-frameshift proteins, and microRNAs [46–52]. These factors function precisely and efficiently in cells to degrade RNA in a timely manner and ensure cell health. When RNAs are released extracellularly to dissolve in water, the factors that induce RNA degradation in the extracellular environment differ from those in the intracellular environment. Extracellular RNAs are readily degraded by ribonucleases released into the environment by various organisms [53–55]. Other biological, physical, and chemical factors, such as hydrolytic enzymes, aqueous solutions, and ions, also play important roles in the degradation of extracellular RNAs [56–60]. If other environmental conditions are known, it is possible to consider inferring the RNA age based on the sequence information of RNA degradation fragments, thereby obtaining information such as the start time of transcription events in fish cells and the time the fish stays in a certain water area in the wild [61].

In the experiment on the degradation of RNA extracted from zebrafish cells in a non-sterile aqueous environment, we measured the samples at four time points using the TapeStation system, which differed from the number of measurements performed using the NanoDrop Spectrophotometer and gel electrophoresis. This discrepancy made it difficult to compare the data obtained by these methods and also rendered the estimate of the RNA half-life relatively rough.

The modelling results showed that dissolved environmental RNAs from zebrafish degraded faster in water containing zebrafish cells than that in water without zebrafish cells (Figure 5(C)). Factors secreted by the live cells and released by disrupted, dying cells may play important roles in the degradation of dissolved environmental RNAs. Regarding the degradation rate of free zebrafish RNA in water in the tubes (Figure 4(C1)), the tubes contained degradation activity factors from the non-sterile environment. However, how these factors affect zebrafish RNA degradation compared to the activity of related factors from zebrafish cells is unknown. Moreover, we made an assumption to explain the dynamic changes in dissolved RNA in the liquid environment of a flask. In this assumption of RNA production and degradation in organisms [43,62–64], RNA release from cells is independent of RNA abundance in the culture medium or nucleic acid-free water, but RNA degradation is dependent on RNA abundance in the culture medium or nucleic acid-free water. Based on this assumption, we used mathematical models to calculate the release and degradation parameters of the dissolved environmental RNA. Clearly, there are certain limitations in using mathematical models originally designed to analyse the dynamic changes of RNA production and degradation in organisms to analyse these dynamic changes of RNA production and degradation in culture medium and nuclease-free water. Therefore, these results need to be verified by measuring the experimental data.

## 4. Conclusion

The present study showed that dissolved environmental RNAs can originate from live and dying fish cells. Environmental fish RNAs dissolved in the water could be detected for more than two months at room temperature, although the most detectable dissolved environmental RNAs might degrade into small RNA fragments. Therefore, dissolved environmental fish RNAs, which are primarily found in the filtrates from the current filtration methods used to detect environmental RNAs in water, may retain biological and ecological information of fish species that once existed or currently existed for a certain period. Although current methods have limitations, the present study may provide information for the development of new methods to measure the dynamics of environmental fish RNAs dissolved in water and for quantitatively analysing RNA released by fish cells. Improved protocols for sequencing the trace amounts of dissolved environmental fish RNAs in water in the field are urgently needed.

## 5. Methods and materials

### 5.1. Culture of zebrafish BRF41 cells and RNA extraction

Zebrafish BRF41 cells (Cell number: RCB0804), derived from the fin fibroblasts of *Danio rerio*, were purchased from the Cell Bank, RIKEN BioResource Research Center, Japan, and cultured in 25 cm^2^ or 75 cm^2^ flasks with the culture medium consisting of 84% Leibovitz’s L-15 medium (Gibco, Thermo Fisher Scientific), 1% 10 mM HEPES (Gibco, Thermo Fisher Scientific), and 15% foetal bovine serum [65]. The culture temperature was set at 33°C. The cells were subcultured every 10 days using 0.25% trypsin (Gibco, Thermo Fisher Scientific) to dislodge the cells from the flask bottoms, followed by washing with phosphate-buffered saline (Gibco, Thermo Fisher Scientific).

RNA was extracted from zebrafish BRF41 cells (passage number = 64) using TRIzol^TM^ reagent (Invitrogen, Thermo Fisher Scientific) following the manufacture’s user guide procedures (Pub. No. MAN0001271 c.0). The BRF41 cells for RNA extraction were grown in 75 cm^2^ flasks or 25 cm^2^ flasks. The cells grew well and the cell confluency was approximately 80%. The extracted RNAs were stored at −80°C for 1 week before being used in the experiments on RNA extracted from zebrafish cells through a 0.45 µm filter (Section 5.2) and RNA degradation (Section 5.5).

### 5.2. Filtration of RNA extracted from zebrafish cells through a 0.45 µm filter

The RNA used in the RNA filtration experiment was zebrafish RNA with a concentration of 65 ng/µL, extracted from zebrafish BRF41 cells with TRIzol^TM^ reagent as described in Section 5.1, and diluted with nuclease-free water. There were three replicates of 65 ng/µL zebrafish RNA and three replicates of the nuclease-free water controls. Two millilitres of the liquid from each replicate of 65 ng/µL zebrafish RNA or the nuclease-free water control were passed through a 0.45 µm filter unit (Sterivex^TM^, EMD Millipore Corporation, Germany), and the filtrates were collected (Figure 1(b)). The selection of a 0.45 µm pore size in this study was based on previous research, in which filters of varying pore sizes were tested for detecting environmental RNA in water. Filters with a 0.45 µm pore size were the most commonly used by researchers. Accordingly, 0.45 µm filters can be considered representative of those used in studies on environmental RNA in water. Furthermore, to estimate the concentrations of dissolved environmental fish RNAs released by zebrafish exfoliated tissue cells in water (Section 5.6.1), the results obtained in this study were combined with those reported by Jo et al. [4], who also used 0.45 µm filters, ensuring consistency between the two datasets. The concentration and fragment sizes of the RNAs in the liquid before and after filtration were measured using an RNA ScreenTape system (Agilent 2200 TapeStation, Agilent Technologies, Inc.) and a NanoDrop ND-1000 Spectrophotometer (Marshall Scientific LLC).

### 5.3. Dissolved environmental RNAs released by live zebrafish cells

In the next phase of the experiment, zebrafish BRF41 cells (passage number = 71) were cultured in 15 mL of culture medium in 75 cm^2^ flasks. The initial number of seeded cells was 6.8 × 10^5^ cells per flask. There were three flasks of zebrafish BRF41 cells cultured in 15 mL of culture medium (designated ‘Cells in Medium’) and three flasks of culture medium without zebrafish BRF41 cells (designated ‘Medium Control’). The zebrafish BRF41 cells for the experiment grew well, with the cell confluency reaching approximately 80%. After subculturing these two groups, 1 mL of culture medium from each flask of the Cells in Medium and Medium Control groups was sampled to measure RNA on days 0, 1, 2, 3, 4, and 5. The entire 1 mL of medium from each sample was passed through a 0.45 µm filter unit, and the filtrates were measured using a High Sensitivity RNA ScreenTape (Agilent 2200 TapeStation, Agilent Technologies, Inc.). The cells in the flasks were observed under a microscope (Olympus IX70) and photographed using the CellSens standard (Olympus). On the day 5, the number of cells in the Cells in Medium flasks was counted using a haemocytometer. The experimental operation was carried out in a sterile environment. The RNA mass in a flask of Cells in Medium was calculated as follows:

RNA mass in a flask = (RNA concentration in Cells in Medium – RNA concentration in Medium Control) × volume of medium in the flask

### 5.4. Dissolved environmental RNAs released by dying zebrafish cells in nuclease-free water

Zebrafish BRF41 cells (passage number = 71) cultured in culture medium were dislodged from the flask bottom using trypsin, rinsed with phosphate-buffered saline, resuspended in 1.5 mL of the culture medium, and then placed the volume of the resuspended culture medium containing 1.0 × 10^6^ cells into a flask filled with 15 ml of nuclease-free water. The number of seeded cells in each flask was 1.0 × 10^6^ cells. There were three flasks of zebrafish BRF41 cells cultured in nuclease-free water (designated ‘Cells in NF’) and three flasks of controls with nuclease-free water and trace culture medium (the amount of which was equal to that used when transferring cells in the Cells in NF groups) but without zebrafish BRF41 cells (designated ‘NF Control’). The RNA in the water of the Cells in NF and NF Control groups was measured on days 0, 1, 2, 3, 4, and 5. One millilitre of nuclease-free water was passed through a 0.45 µm filter unit, and the filtrates were collected. The concentration and fragment sizes of the RNAs in the filtrates were measured using a High Sensitivity RNA ScreenTape. The cells in the flasks were observed under a microscope (Olympus IX70) and photographed using the CellSens standard (Olympus). Every day, the number of suspended cells was counted using a haemocytometer. We assumed that the cells attached to the bottom of the flasks on day 6 were live cells, and the cells that did not attach to the bottom of the flasks within 6 days were dying cells. The experiments were performed in a sterile environment. The RNA mass in a flask of Cells in NF was calculated as follows:

RNA mass in a flask = (RNA concentration in Cells in NF – RNA concentration in NF Control) × volume of nuclease-free water in the flask

### 5.5. Degradation of RNA extracted from zebrafish cells in a non-sterile aqueous environment

A 61-day experiment was conducted to study the degradation of the RNA extracted from zebrafish cells in water, focusing on the self-degradation process and degradation activity factors in a non-sterile environment. RNA extracted from zebrafish BRF41 cells as described in Section 5.1, was diluted with tap water to a concentration of 65 ng/µL. A 100 µL aliquot of this RNA solution was placed into 1.5 mL centrifuge tubes at room temperature (25 °C). To prevent evaporation, the centrifuge tubes were capped unless they were being used for sampling. Three centrifuge tubes containing RNA at a concentration of 65 ng/µL and three centrifuge tubes containing only tap water (controls) were prepared. RNA concentrations were assessed using the RNA ScreenTape system every 10 days, normal gel electrophoresis every 5 days, and a NanoDrop ND-1000 spectrophotometer every 2 days. The conditions for the normal gel electrophoresis were as follows: 100 volts, 25 minutes of running time, 2.5% agarose (Nippon Gene Co., Ltd, Japan), 1×TAE buffer, and the ladder of Gene Ladder Wide 1 with the range of 100–20000 bp (Nippon Gene Co., Ltd, Japan). The electrophoresis was carried out using the Mupid®-2plus (Takara Bio Inc, Japan) and visualized using a UV-trans-illuminator of ATTO Printgraph Type GX (ATTO Corporation, Japan). The sampling operation was carried out in a non-sterile environment, and the tubes containing the zebrafish BRF41 cell RNAs were also placed in this non-sterile environment. The use of tap water in the experiment was justified for the following reasons: tap water is not considered sterile water, can be used in experiments to measure environmental RNA in water in zebrafish tanks [4], is a resource that is readily available resource, and has a quality (tap water quality data can be obtained through open channels, such as http://www.jwwa.or.jp/mizu) that is more stable than that of other water resources, such as ponds, lakes, and rivers.

### 5.6. Estimation of the release and degradation of dissolved environmental RNA from zebrafish cells using mathematical models

#### 5.6.1. Estimation of dissolved environmental RNA levels in fish tanks

Based on the current study where zebrafish cells in nuclease free water is arguably comparable to the experiment by Jo et al. [4], which describes zebrafish exfoliated tissue cells in water as comparable to zebrafish in tanks, the experimental data in the prior study is combined with the current study to estimate the level of zebrafish derived environmental RNA in tanks. In the study by Jo et al. [4] on environmental RNA released by zebrafish in tanks, the amount of the mitochondrial DNA gene CytB from DNA extracted from a 300 mL water sample on day 0 was 1203.5–8516.9 copies/PCR (Supporting Information section [4]). This DNA was contributed by the cells shed from the zebrafish. Each adult zebrafish cell contains 5500 copies of its mitochondrial genomes [66]. The DNA in the cells in the water may have been partially degraded or lost and could not be collected by the filter. For example, there were only 1203.5 copies of the CytB gene in the cells contained in a 300 mL water sample. We assumed that each zebrafish cell in the water sample contained 1203.5 ~ 5500 copies of the CytB gene. Dividing the amount of CytB gene (1203.5 ~ 8516.9 copies) in a 300 ml water sample by the amount of CytB gene (1203.5–5500 copies) in each zebrafish cell in the water, we estimated that each fish tank contained 0.7–23.7 cells/L (or 0.22–7.1 cells per 300 mL).

#### 5.6.2. Estimation of the rates of release and RNA degradation of dissolved environmental RNA from zebrafish cells

In the experiment on the degradation of RNA extracted from zebrafish cells in a non-sterile aqueous environment, the RNA degradation rates were measured directly. However, in the experiments on dissolved environmental RNAs released by live and dying zebrafish cells, the RNA mass, determined by RNA release and degradation rates, was measured indirectly. In cells, transcription rates and RNA degradation rates could be estimated from time series RNA abundance data by using the mathematical modelling [67,68]. Similarly, using the model outlined below, RNA abundance data were applied to estimate RNA release rates and RNA degradation rates in the medium or water.

The basic unit of the model is based on the assumption of RNA production and degradation [43,62–64]: under stable conditions, RNA release from cells is assumed to be independent of RNA abundance in the medium or water, but RNA degradation depends on RNA abundance in the medium or water. The change in RNA abundance was calculated as the amount of RNA released minus the amount of degradation, as described by the following equations:

(1) $$\frac{\mathrm{dN}}{\mathrm{dt}}=r-\mathrm{\lambda N}$$

(2) $$N_{t}=\frac{\lambda N_{0}-r}{\lambda }e^{-\mathrm{\lambda t}}+\frac{r}{\lambda }\;\left(\mathrm{when}\;\lambda >\;0\right)$$

(3) $$N_{t}=\mathrm{rt}+N_{0}\;\left(\mathrm{when}\;\lambda =\;0\right)$$

where *N* is the RNA mass, *t* is time, *r* is the RNA release rate coefficient (e.g. the producing rate coefficient of the dissolved environmental RNA), *λ* is the degradation rate coefficient, *N*_*t*_ is the RNA abundance at time t, and *N*_*0*_ is the RNA abundance at time 0.

However, as it may be difficult to directly use Equation 2 or Equation 3 if the experimental time period is extended and various factors influence the results, we analysed the data over a shorter time period. Therefore, we set several continuous sampling time points as estimation units, and Equations 2 and 3 were applied to analyse the data within each unit. After completing the analysis of the previous estimation unit, the next estimation unit was shifted to the subsequent sampling time point.

Within the estimation units, the predicted RNA abundance *N*_*t*_ was calculated using Equations 2 and 3 at each sampling time point. If more than one estimation unit covered a sampling time point, the mean predicted RNA abundance at that time point was calculated as follows:

(4) $$\mathrm{Mean}\_\mathrm{RNA}\_\mathrm{abundanc}e_{t}=\frac{\sum N_{t}}{c}$$

where *Mean_RNA_abundance*_*t*_ is the mean of the predicted RNA abundance at time *t*, *N*_*t*_ is the predicted RNA abundance at time *t* in an estimation unit, and *c* is the number of estimation units covering the sampling time point. Once the mean predicted RNA abundance at all sampling time points was obtained, the fitting index between the predicted RNA abundance and the experimental data was calculated. The R-squared and median absolute percentage error (MdAPE) values were used as the fitting indices.

The RNA release rate was calculated as follows:

(5) $$\mathrm{Nne}t_{t}=\mathrm{rt}+\mathrm{Nne}t_{0}$$

(6) $$\mathrm{RNA}\_\mathrm{Releas}e_{t}=\mathrm{Nne}t_{t}-\mathrm{Nne}t_{t-1}$$

(7) $$\mathrm{Mean}\_\mathrm{RNA}\_\mathrm{Releas}e_{t}=\frac{\sum RNA\_R\mathrm{eleas}e_{t}}{c}$$

(8) $$\mathrm{Cumulative}\_\mathrm{RNA}\_\mathrm{Releas}e_{t}=\sum_{t=0}^{s}\mathrm{Mean}\_\mathrm{RNA}\_\mathrm{Releas}e_{t}$$

(9) $$\mathrm{RNA}\_\mathrm{Release}\_\mathrm{Rat}e_{t}=\frac{C\mathrm{umulative}\_RNA\_R\mathrm{eleas}e_{t}}{t}$$

where *t* is time, *Nnet*_*t*_ is the amount of RNA without degradation at time *t*, *r* is the RNA release rate coefficient, *Nnet*_0_ is the amount of RNA without degradation at time 0, *RNA_Release*_*t*_ is the RNA release amount at time *t*, *Nnet*_*t-1*_ is the RNA amount without degradation at time *t*-1, *Mean_ RNA_Release*_*t*_ is the mean RNA release amount at time *t*, *c* is the number of simulation units in which the sampling time point at time *t* is covered, *Cumulative_ RNA_Release*_*t*_ is the cumulative RNA release amount during time *t*, *s* is a specified point in time, and *RNA_Release_Rate*_*t*_ is the RNA release rate at time *t*.

The RNA degradation rate was calculated as follows:

(10) $$\mathrm{Degradatio}n_{t}=\mathrm{Nne}t_{t}-N_{t}$$

(11) $$\mathrm{Mean}\_\mathrm{Degradatio}n_{t}=\frac{\sum D\mathrm{egradatio}n_{t}}{c}$$

(12) $$\mathrm{Cumulative}\_\mathrm{Degradatio}n_{t}=\sum_{t=0}^{s}\mathrm{Mean}\_\mathrm{Degradatio}n_{t}$$

(13) $$\mathrm{Degradation}\_\mathrm{Rat}e_{t}=\frac{C\mathrm{umulative}\_D\mathrm{egradatio}n_{t}}{t}$$

where *Degradation*_*t*_ is the amount of RNA degradation at time *t*, *Nnet*_*t*_ is the amount of RNA without degradation at time t, *N*_*t*_ is the RNA abundance at time t, *Mean_Degradation*_*t*_ is the mean RNA degradation amount at time *t*, *c* is the number of estimation units in which the sampling time point at time *t* is covered, *Cumulative_Degradation*_*t*_ is the cumulative RNA degradation amount at time *t*, *s* is a specified time point, and *Degradation_Rate*_*t*_ is the RNA degradation rate at time *t*.

The simulation processes were translated into the C++ language (Code S1).

## Data Availability

An early version of this article was upload to a non-commercial preprint server [69]. The datasets generated and/or analysed during the current study are included in this published article and its supplementary information files. Other data (such as program operation results, etc.) will be provided upon reasonable request.
